# Dual sensory loss: development of a dual sensory loss protocol and design of a randomized controlled trial

**DOI:** 10.1186/1471-2318-13-84

**Published:** 2013-08-13

**Authors:** Hilde L Vreeken, Ger HMB van Rens, Sophia E Kramer, Dirk L Knol, Joost M Festen, Ruth MA van Nispen

**Affiliations:** 1 Department of Ophthalmology, VU University Medical Center, Amsterdam, The Netherlands; 2 EMGO+ Institute for Health and Care Research, VU University Medical Center, Amsterdam, The Netherlands; 3 Department of Opthalmology, Elkerliek Hospital, Helmond, The Netherlands; 4 Department of Otolaryngology, VU University Medical Center, Amsterdam, The Netherlands; 5 Department of Epidemiology and Biostatistics, VU University Medical Center, Amsterdam, The Netherlands

**Keywords:** Dual sensory loss, Visual impairment, Hearing loss, Elderly, Low vision rehabilitation, Hearing aids, Communication, Intervention, RCT

## Abstract

**Background:**

Dual sensory loss (DSL) has a negative impact on health and wellbeing and its prevalence is expected to increase due to demographic aging. However, specialized care or rehabilitation programs for DSL are scarce. Until now, low vision rehabilitation does not sufficiently target concurrent impairments in vision and hearing. This study aims to 1) develop a DSL protocol (for occupational therapists working in low vision rehabilitation) which focuses on optimal use of the senses and teaches DSL patients and their communication partners to use effective communication strategies, and 2) describe the multicenter parallel randomized controlled trial (RCT) designed to test the effectiveness and cost-effectiveness of the DSL protocol.

**Methods/design:**

To develop a DSL protocol, literature was reviewed and content was discussed with professionals in eye/ear care (interviews/focus groups) and DSL patients (interviews). A pilot study was conducted to test and confirm the DSL protocol. In addition, a two-armed international multi-center RCT will evaluate the effectiveness and cost-effectiveness of the DSL protocol compared to waiting list controls, in 124 patients in low vision rehabilitation centers in the Netherlands and Belgium.

**Discussion:**

This study provides a treatment protocol for rehabilitation of DSL within low vision rehabilitation, which aims to be a valuable addition to the general low vision rehabilitation care.

**Trial registration:**

Netherlands Trial Register (NTR) identifier: NTR2843

## Background

As a result of the aging of the Western population, impairments of hearing and vision caused by age-related degeneration of the senses (e.g. presbyacusis and macular degeneration) are expected to increase rapidly [1,2]. A relatively large number of older adults suffer from concurrent impairments in hearing and vision, also known as dual sensory loss (DSL) [3-8]. Among older adults, the prevalence of DSL ranges from 4.7 - 9.7% in the general population and from 20-45% in those in nursing homes, hospitals and low vision rehabilitation [1,9-13].

DSL has a detrimental effect on a patient’s health and well-being in relation to, for example, communication, social participation, depression, cognition, functional disability, health-related quality of life, self-rated health and mortality [3-5,13-22]. Presumably, DSL also affects communication partners. Studies on single sensory loss show that communication partners experience caregiver burden, depressive symptoms, disability, tension in the relationship and show increased reliance on informal care [23-27]. Besides support from friends and family, sensory impaired patients also rely on support from community services [26,28] and sensory loss is associated with high societal costs [29-31].

Two main issues affect the lives of DSL patients. First, DSL impairs proper use of hearing aids. The complexity and small size of hearing aids makes handling and maintenance difficult for DSL patients. Examples of difficulties are inserting the hearing aid or ear mold in the ear, seeing the controls, or replacing batteries (glare from reflective surfaces may interfere with seeing the battery polarity) [32]. Therefore, concurrent visual impairment could (in addition to other age-related discomforts such as tactile sensitivity and manual dexterity) impede the handling and maintenance of hearing aids. This may result in misuse and/or underuse of hearing aids [32]. Studies among hearing-impaired older adults reported a low rate of hearing aid use [33-35]. Although older adults with DSL are in great need of amplification (because of decreased lip-reading ability due to visual impairment) [32], many patients experience problems with handling hearing aids or do not use them at all despite their well-known benefits on e.g. on quality of life [36]. Since DSL patients are less able to compensate with visual cues, they depend heavily on their (aided) hearing. Therefore, improper and/or non-use of hearing-aids may lead to result in a detrimental effect on health.

Second, DSL impairs communication as both good visual and hearing acuity enhance speech understanding. In DSL patients, age-related hearing loss reduces the ability to discriminate speech. In turn, a visual impairment reduces the perception of visual cues supporting speech understanding, such as looking at the speaker’s face/mouth and other bodily movements and gestures [32,37-40]. Although use of hearing aids has a positive effect on communication, problems persist in common noisy listening situations where hearing aids are inadequate [41]. When communication frequently fails, negative experiences can lead to avoidance of conversations and so-called ‘communication break downs’. These break downs are highly distressing and can cause feelings of loneliness, social isolation and depression [42-45].

Although DSL patients would benefit from rehabilitation to cope with these problems, integrated rehabilitation of DSL is scarce. In current practice, rehabilitation of sensory impairments in the aging population is divided into separate rehabilitation services for impairments in vision (low vision rehabilitation centers) and in hearing (audiology centers and hearing aid providers). Concurrent hearing impairment could affect the success of low vision rehabilitation [12] and vice versa. Moreover, healthcare providers do not automatically deal with impairment of the ‘other sense’, which may lead to less effective rehabilitation. Therefore, Saunders & Echt (2007) recommended to combine these two independent services [46]. In fact, the development and systematic evaluation of multidisciplinary integrated rehabilitation of DSL in older adults (i.e. communication training in which communication partners are involved) is considered one of the most urgent research needs in health care [46-48].

This paper reports on the development of a ‘Dual Sensory Loss-protocol’ (DSL protocol) designed for occupational therapists (OTs) working in the field of low vision rehabilitation, which focuses on maximal use of remaining hearing and vision. The protocol presents an integrated treatment of DSL for older adults within low vision rehabilitation. In addition, the design and methodology of a randomized controlled trial (RCT) to evaluate the effectiveness and cost-effectiveness of this protocol is described.

## Methods/Design

### DSL protocol

#### Development

In the development of the DSL protocol, literature was reviewed, and patients and professionals were consulted. First, the literature was reviewed on the topic of rehabilitation of DSL, and on existing interventions or recommendations on rehabilitation of DSL [42,46,49-57] and audiological rehabilitation [41,58]. Results of the literature review and content of the protocol were discussed in interviews and two focus group discussions with professionals in low vision and audiological rehabilitation. Professionals participating in the focus groups were: two OTs, a social worker, two clinical physicists and three psychologists (two from the field of low vision and one from audiological rehabilitation) and an audiologist. These professionals discussed the design (e.g. manual, checklist, use of a hand-out card with tips and recommendations for communication partners) and content of the DSL protocol (e.g. the importance of raising awareness, provision of information on hearing assistive devices) and also came up with specific suggestions (e.g. referral to audiological centers, social work or peer groups). In these discussions, it was decided that the DSL protocol would be a new intervention on top of usual care of low vision, after remaining eyesight is optimized as much as possible.

However, because professionals may have some (work-related) bias, DSL patients and their communication partners were also consulted [58]. Three DSL patients (aged 50+ years) and one patient’s partner were interviewed during home visits. The patients were invited to participate by the Dutch Foundation for the Deafblind and by a participating low vision rehabilitation center (Bartiméus). In all patients the cause of deaf-blindness was Usher syndrome. Patients were asked what problems they generally encountered, and also provided advice for new patients; e.g. they recommended patients that patients consult other patients for help and also involve the family in patient care.

A draft of the DSL protocol was sent to all professionals involved; in two feedback rounds, they were asked to provide comments/suggestions on the draft.

The DSL protocol provides information on DSL. However, because of the aim to implement the protocol in low vision rehabilitation centers, it also focuses on the gap in knowledge related to audiology and rehabilitation in that field.

#### Topics described in the DSL protocol

In the DSL protocol, rehabilitation is divided into three chapters (Chapter 1: Hearing aids; Chapter 2: Optimal use of the senses; living environment & hearing assistive devices; Chapter 3: Communication and coping with DSL). Chapter 1 of the DSL protocol includes information on audiology and the benefits/limitations of hearing aids, and also focuses on the proper use/maintenance of hearing aids. The chapter starts by informing the patient/communication partner about both vision and hearing loss to raise recognition, awareness, knowledge and understanding of sensory impairments. Patients/communication partners are informed about the benefits/limitations of hearing aids in order to develop realistic expectations and, for the communication partner to gain understanding of the situation. Note that care must be taken in addressing unrealistic expectations, as too low expectations could demotivate or discourage patients from using hearing aids [59].

Then, the DSL protocol focuses on the proper and optimal use of hearing aids. Although correct use of hearing aids is a prerequisite, it cannot be presumed. Hearing aid users benefit from training [54]; training in how to handle, manipulate, insert and remove hearing aids and test batteries enhances hearing aid use and maintenance. However, older adults may need additional instruction time to acquire these skills [60]. Because of the expected difficulties related to the visual impairment and limited training from hearing aid suppliers, exercises to teach these procedures to DSL patients and/or proxies are included in the first chapter of the DSL protocol. OTs teach and train patients (or communication partners) how to handle and maintain hearing aids with the use of low vision devices (e.g. stand magnifiers or CCTV) [46].

In the second chapter, the DSL protocol focuses on optimal use of the senses by improvement of the living environment in relation to lighting, acoustics and proximity, and the use of low vision and hearing assistive devices [46,61,62]. To improve understanding of speech, the OT advises to make minor adaptations to optimize the living environment to improve visibility and audibility (if required). For example, an OT may, for example, recommend the patient to reduce the distance between communication partners (proximity) to improve visibility and audibility [52]. To enhance acoustics, the OT may, for example, recommend to reduce background/room noise and to reduce reverberation with sound-absorbent furnishings such as heavy curtains, carpeting and cushions [46]. Depending on the situation, OTs may also advise patients on acoustics, lighting and proximity. Subsequently, OTs provide advice and information on assistive devices for hearing and vision, and about the interconnectivity of the devices.

Communication and coping with DSL is the focus of the third chapter; it stimulates use of communication strategies (patients and communication partners) and social participation, it also discusses problems with energy/fatigue, and provides information on peer support. Communication difficulties and decreased social activity of DSL patients have a negative impact on wellbeing [45]. Use of effective communication strategies such as seeing the speaker (use of facial cues by face-to-face orientation and visual attention) might enhance communication in difficult situations [63]. These strategies focus on optimizing auditory-visual speech perception by enhancing face-to-face communication, effects of high visual contrast, glare, illumination and distance on visual-speech perception [37]. Although the severity of the visual impairment of DSL patients affects their ability to ‘see the speaker’, simulations have shown that even severely visually impaired persons are able to use visual cues up to some extent for speech reading [37,39,63] and that DSL patients found learning new strategies useful [56]. Parts of an existing and effective communication training program for hearing-impaired older patients and their hearing communication partners, developed by Kramer et al. (2005) have been incorporated in the DSL protocol. In this training, older adults with hearing loss only (and their hearing communication partners) learned to use communication strategies [41]. Others have also proposed involving communication partners in the training of communication strategies [55]. Despite the fact that DSL patients regularly experience communication difficulties, communication partners are often unaware of these problems. OTs address communication difficulties and teach DSL patients and communication partners to use effective communication strategies in addition to hearing aids, which may also improve their quality of life. Subsequently, OTs encourage the patient to bring these newly learned strategies into practice, and to participate in social activities that they previously enjoyed, but ceased because of communication difficulties induced by DSL [49]. Thirdly, another problem confronting DSL patients is fatigue. DSL patients often feel exhausted, especially in communication, when concentration and effort is required for listening and understanding [56]. OTs address the problem of fatigue and discuss management of the energy balance. Finally, OTs provide information on patient organizations and peer groups which can provide some support.

#### Two parts of the DSL protocol

The protocol is divided into two parts: i) a comprehensive guidebook for the professional, and ii) checklists for each patient. The guidebook provides background information, materials and comments on each exercise. Materials include a CD, DVD, large printed handouts (font 14, light-yellow colored matte/non-glossy paper), information on patient organizations, a large printed picture of a loop system sign, information on financial compensation for assistive devices, a handout with instructions on hearing aid use and maintenance, and handouts with communication strategies for patient and communication partners.

The second part of the protocol consists of checklists for each patient with exercises and instructions, which follow the three chapters of the guidebook. Items of the checklist are:

Introduction

1) Discuss goal and design of treatment

2) Check severity of vision and hearing loss

3) Check available low vision and hearing aids

4) Check if the patient has comorbidity

5) Create awareness with the patient of his/her dual sensory loss

6) Create awareness of the communication partner about dual sensory loss (CD)

Chapter 1: Hearing aids

7) Give information on hearing aids and discuss realistic expectations

8) Discuss experiences and problems with hearing aids

9) Check working of hearing aid, batteries and tube

10) Check amplification of hearing aid

11) Check replacement of hearing aid and ear mold

12) Check visibility of hearing aid, advise on use of low vision devices if necessary

13) Check the possibilities of the hearing aid, such as settings/modes and programs

14) Check skills in hearing aid use and manipulation of controls

15) Check batteries and battery replacement

16) Mark hearing aid

17) Dry clean ear mold

18) Wet clean ear mold

*Chapter 2: Optimal use of the senses: living environment* &*hearing assistive devices*

19) Lighting for speech reading

20) Advice on acoustics for speech intelligibility

21) Use of loop systems in public buildings

22) Use of subtitles and spoken subtitles

23) Check whether the patient would benefit from other hearing assistive devices

Chapter 3: Communication and coping with DSL

24) Address problems related to fatigue and energy balance

25) Use of communication strategies by the patient (handout)

26) Use of communication strategies by communication partner (handout)

27) Discuss the use of communication strategies on the basis of propositions

28) Address DSL (vision and hearing impairment) to the speaker

29) Ask speaker for clarification

30) Discuss communication strategies using specific questions

31) Provide information on patient organizations and peer groups

Home assignment

a) Watch the DVD ‘Hearing and being heard’ on the use of communication strategies, together with the communication partner. Discuss the situations on the DVD using questions.

b) Attend a social activity, to apply the new skills and communication strategies.

The OT will go through the checklist in 3–5 sessions at the patient’s home; participation of the communication partner is strongly recommended. The sessions are divided in two parts of 30 minutes each, separated by a break of 15 minutes. This takes into account, the length of the sessions, as well as the rapid fatigue and decreased ability of older adults with DSL to concentrate for longer periods of time. The exact number of sessions needed to go through the checklist depends on the abilities/needs of the individual patient. We anticipate the (valuable) participation of communication partners, since communication is interactive and relies on the conversational abilities of both communication partners.

#### Training of OTs and pilot-study

To use the DSL protocol, OTs need training. For the current RCT to test the effectiveness of the DSL protocol, OTs from two participating rehabilitation institutions were offered a one-day training in the DSL protocol on October 18th, 2011. The first part of the training was provided by an audiologist and involved background information on audiology. Subsequently, a hearing aid provider gave instructions on the use and maintenance of hearing aids, exercises on hearing aid use, and gave information and a demonstration on hearing assistive devices. In the second part of the training, a speech therapist taught the OTs how to teach patients and proxies to use adequate communication strategies, e.g. with the use of role play. In role play, special glasses and earmuffs were used to simulate vision and hearing loss of DSL patients. The training was filmed and later on three other OTs were later trained with using this film as part of their training.

In a limited pilot study (before the start of the RCT) OTs went through the DSL protocol with a DSL-patient in a real-life situation. The results of this pilot study were evaluated in three meetings with the OTs and, subsequently, a few clarifying adaptations were made to the protocol.

### Design and methodology of the RCT

#### Study design

**Figure 1:**
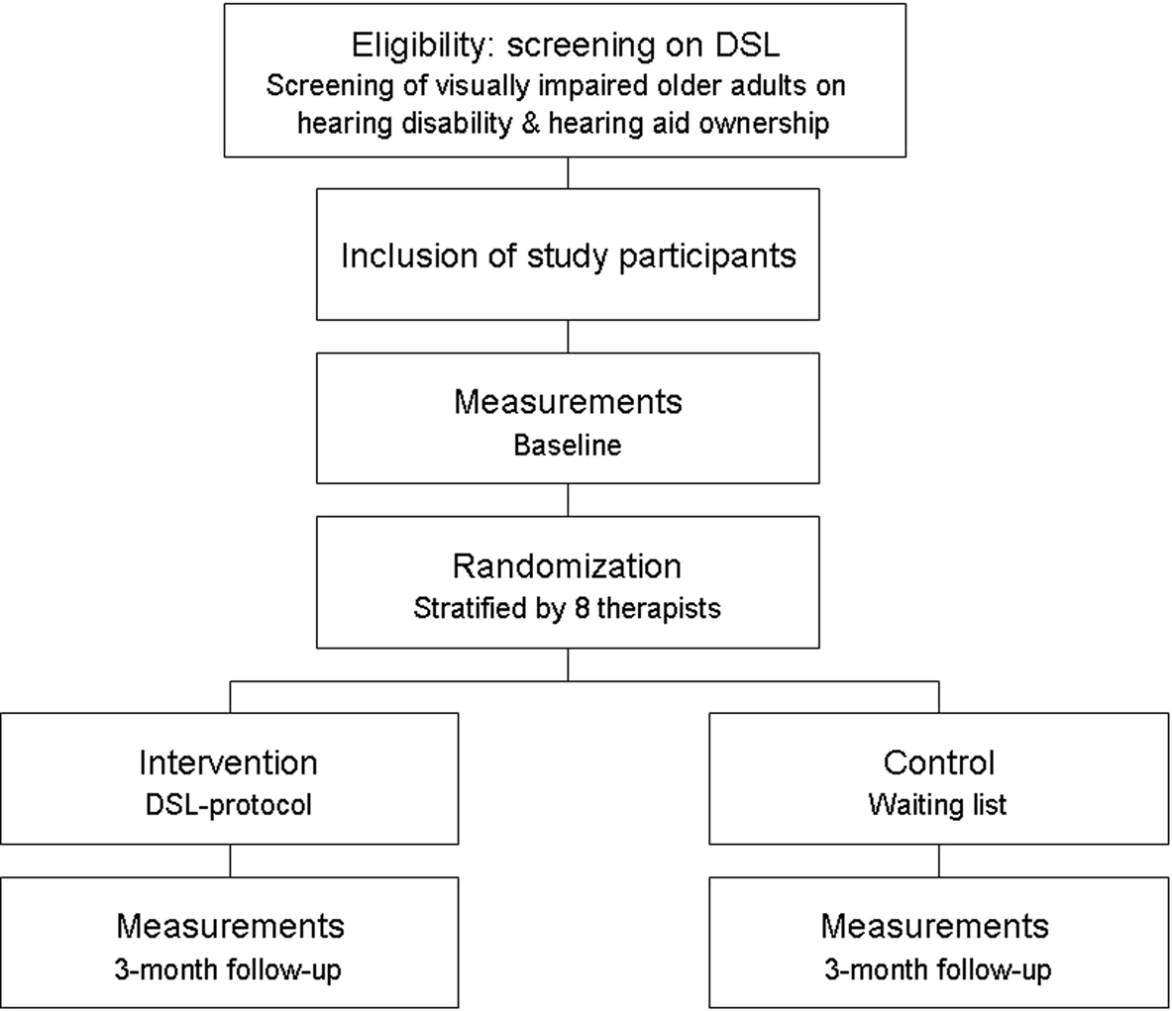
Design of the trial.

A two-armed international multicenter parallel RCT will be conducted to evaluate the health effectiveness and cost-effectiveness of the DSL protocol from a societal perspective in low vision multidisciplinary rehabilitation institutions in the Netherlands and Belgium. The design of the trial is shown in Figure 1.

The study is approved by the Medical Ethical Review Committee of the VU University Medical Center (the Netherlands) and the Ethical Committee of University Hospitals UZ/KU Leuven (Belgium) according to the principles of the Declaration of Helsinki. The trial is registered at the Netherlands Trial Register (http://www.trialregister.nl, identifier: TC = 2843).

#### Study population

Prior to the trial, patients of low vision rehabilitation were screened for eligibility. A short, large-print questionnaire on hearing problems and hearing aid ownership was sent to all patients (aged >50 years) who received low vision rehabilitation between January 1 and October 31, 2012. Addresses were derived from the patient information databases of the rehabilitation institutions. After two weeks, patients who did not fill in the questionnaire were telephoned about the questionnaire and in case the patient was not able to fill in the questionnaire due to low vision, the questions were administered orally by telephone. More details on the screening and the results of the screening are published elsewhere [11].

Patients who experience hearing problems and are in the possession of a hearing aid will be invited to participate in the RCT. Hearing aid owners will be invited to participate because hearing aids, and making optimal use of hearing aids, are an important part of the DSL protocol. In addition, by selecting hearing aid owners with hearing problems by using information from the questionnaire, patients with both objective hearing loss (reimbursement of hearing aids from a hearing loss of at least 35 dB) and self-reported hearing disability will be selected; these patients are expected to be motivated and the most suitable for rehabilitation [48]. Moreover, due to time constraints, it is not possible to wait for patients to fit hearing aids in order to be able to take part in the trial; this procedure may take months due to the required testing period and possible reimbursement.

Written information will be sent to eligible patients and informed consent will be signed prior to inclusion. It is expected that about 1500 patients need to be screened to include 124 eligible participants in the trial. Patients who are unable to comprehend or respond to questions due to cognitive impairment, or have insufficient knowledge of the Dutch language, will be excluded from the study. Table 1 summarizes the inclusion/exclusion criteria.

**Table 1:** Inclusion and exclusion criteria for the randomized controlled trial

***Inclusion***	***Exclusion***
Age ≥ 50 years	Cognitive deficits
Visual impairment (low vision rehabilitation)	Deaf persons
Hearing impairment:	Insufficient knowledge of the Dutch language
• Self-reported hearing problems*	
• Hearing aid ownership (mean pure tone thresholds at 1000, 2000 and 4000 Hz >35 dB the Netherlands, and >40 dB Belgium)	

*See Vreeken et al. 2013 [11].

#### Randomization

After baseline measurements, participants will be randomly allocated (stratified by therapist) to either the intervention or control group, by means of computer-generated tables. Trained OTs employed by the participating low vision multidisciplinary rehabilitation institutions in the Netherlands and in Belgium will provide therapy for older adults with DSL and their communication partner (i.e. the intervention group) according to the newly developed DSL protocol. To prevent unequal distributions of participants in the control and intervention groups among OTs, participants will be stratified within working areas of OTs before randomization resulting in eight strata, one for each of the eight participating OTs (four OTs from Bartiméus, two OTs from Visio and two OTs from Blindenzorg Licht en Liefde). Block randomization will be performed in blocks of two to ensure equal group sizes in each stratum. Randomization will be performed by an independent researcher using a computer-generated allocation scheme so that the researcher who will analyze the data is blinded (trained research assistants will enter all data into a laptop computer using codes). All participants will be informed about the allocation. In case a participant is allocated to the treatment group, an email will be sent to inform the designated OT. Treatment will take place in the participant’s home in 3–5 appointments, depending on the individual needs of the participant and/or proxy. All participants have received low vision rehabilitation and hearing aid fitting before entering the study. Using a waiting list procedure, participants in the control group will be offered treatment after 3-month follow-up measurements.

#### Data collection

Measurements will include face-to-face structured interviews and will be performed by trained research assistants in a quiet room in the home of the participant at baseline and at 3-months follow-up. Data collection will take about two hours per measurement. The software program Blaise Enterprise 4.7 (Heerlen, the Netherlands) will be used to directly enter data into laptop computers. Communication partners will be asked to fill in a written questionnaire at baseline and at 3-months follow-up. To measure the ability of understanding speech in noise (speech-reception-threshold in noise), a speech-in-noise test developed by Smits et al. (2013) will be included in the baseline measurements [64]. In the present study, the test will be programmed on laptop computers and performed with headphones (Speedlink Medusa). Digit triplets, uttered in Dutch, will be presented in noise. The participant will repeat the triplet he/she has heard out loud, after which the research assistant will type the digits into the laptop computer and the next digit triplet will be presented to the participant.

Three months after data collection, all participants (including non-compliant participants) will be approached for follow-up measurements.

### Outcome measures

#### DSL patient

The effects of the intervention will be evaluated in terms of hearing aid use and satisfaction, communication (primary outcomes), coping, psychosocial health and perceived quality of life (secondary outcomes) using several validated questionnaires (Table 2).

**Table 2:** Measurements assessed in DSL patients and their communication partners at baseline and 3-month follow-up

	**Baseline**	**3-month follow-up**
**DSL patients**		
*Primary outcome measures*		
Hearing aid fitting, hearing aid use and satisfaction	X	X
Difficulties with hearing aid use and maintenance	X	X
Change in hearing aid use (IOI-HA) [65,66]		X
Change from communication strategies (IOI-AI) [67]		X_i_
Communication (CPHI-communication strategies) [68]	X	X
*Secondary outcomes*		
Health (subjective health)	X	X
Health related quality of life (EuroQol-5 Dimensions (EQ-5D)) [69]	X	X
Coping with hearing impairment (CPHI-personal adjustment) [68]	X	X
Vision-related quality of life (LVQOL) [70,71]	X	X
Loneliness (Loneliness scale) [72,73]	X	X
Fatigue (FAS) [74]	X	X
Participation (parts of D-AI-interpersonal interactions and relationships) [75]	X	X
Autonomy (PAQ) [76]	X	X
Evaluation of intervention		X_i_
*Covariates*		
Patient characteristics (e.g. age, gender, education, living arrangement)	X	
Disability characteristics (eye condition, VA, self-perceived vision (VFQ25-general vision subscale [77]), hearing condition, hearing loss (speech-reception-threshold in noise))	X	
Cognition (6-item screener MMSE) [78]	X	
Depressive symptoms (CES-D) [79]	X	X
Major life events between baseline and follow-up		X
**Communication partners**		
*Primary outcomes*		
Coping with hearing loss (HHDI ‘reactions of others’ scale) [80]	X	X
Change from hearing aid use (IOI-HA-SO) [67]		X
Change from communication strategies (IOI-AI-SO) [67]		X_i_
Quality of life (CarerQoL) [81]	X	X
Evaluation of intervention		X_i_
*Secondary outcomes*		
Chronic fatigue (FAS) [74]	X	X
Depression (CES-D) [79]	X	X
Health (subjective health, EQ-5D) [69]	X	X
*Covariates*		
Demographic characteristics (e.g. age, gender)	X	
Relationship with patient (sort and quality of relation)	X	X
Self-efficacy (G-SES) [82]	X	X
**Costs**		
Healthcare use (iMCQ) [83]	X	X
Intervention costs (occupational therapists, travel costs, time communication partner)		X_i_
Costs informal care (SF-HLQ, time spent on care for communication partner)	X	X
Proxy: Travel time and expenses	X	
Proxy: Time spent on care giving for communication partner	X	X

X_i_ Assessed in intervention group only.

#### Primary outcome measures

*Difficulties with hearing aid use and maintenance* will be measured with questions on problems with hearing aid use and maintenance, e.g. “How difficult is it for you to insert your hearing aid?” or “How difficult is it for you to change your hearing aid batteries?”. Questions on *hearing aid fitting, hearing aid use and satisfaction* will be based on the Questionnaire for evaluation of hearing aid fitting, e.g. hours of hearing aid use, use of hearing aids in different situations, and appreciation of hearing aids [84]. *Self-reported change from hearing aids* is measured with the Dutch version of the widely used International Outcome Inventory for hearing aids (IOI-HA) [65,66,85]. The Communication Profile for the Hearing Impaired (CPHI) is an instrument to measure coping behavior related to hearing impairment and is divided into two domains: ‘Communication Strategies’ and ‘Personal Adjustment’ [68]. The ‘Communication Strategies’ domain of the Dutch 35-item version of CPHI will be used to measure *Communication* (coping behavior in communicative situations) and consists of three subscales: ‘Maladaptive Behavior’; ‘Verbal Strategies’ and ‘Non-verbal Strategies’ [68]. *Self-reported change from communication strategies* reported by the participant is measured with the Dutch version of the International Outcome Inventory for alternative strategies (IOI-AI) [67]. Both measures have been used for evaluation of communication programs by, e.g. Kramer et al. (2005) and Hickson et al. (2005) [41,48]).

#### Secondary outcome measures

Secondary outcomes will be coping, quality of life, health, fatigue, loneliness, participation and autonomy. First, the domain ‘Personal Adjustment’ of the CPHI will be used to assess change in *adjustment to hearing loss* and consists of three subscales: ‘Self-Acceptance’, ‘Acceptance of Loss’ and ‘Stress & Withdrawal’ [68]. Second, the Low Vision Quality Of Life (LVQOL) questionnaire is used to assess *vision-related quality of life outcomes* of participants [70,71]. The LVQOL consists of four scales: Basic aspects of vision, vision-related Mobility, Adjustment to vision loss, Reading and fine work. *Health* was measured with an item on subjective health and with the Euroqol 5 Dimensions (EQ-5D) questionnaire to measure health status [69]. *Fatigue* is assessed with the Fatigue Assessment Scale [74]. To measure *participation*, a number of items of the Dutch ICF Activity Inventory will be selected from the participation domain ‘Interpersonal interactions and relationships’, e.g. with regard to communication and understanding of DSL [75]. Furthermore, an item on withdrawal from social activities was included: “Are there any activities you withdraw from because of your dual sensory impairment?”. *Autonomy problems* will be assessed with the 9-item version of the Patient Autonomy Questionnaire (PAQ) [76]. Feelings of *emotional and social loneliness* will be measured with the 11-item Loneliness Scale [72]. Examples of the 11 items of this scale will be “I miss heaving a really close friend” (emotional loneliness) and “I can call on my friends whenever I need them” (social loneliness). The DSL protocol was evaluated with the questions: “Are you satisfied with the advice of the OT?”, “Would you recommend the DSL protocol to other DSL patients?”, “Are you satisfied with the treatment?” and “Are you satisfied with the results?”.

#### Independent variables

In addition, *patient* and *disease characteristics* will be assessed. Information on age, gender, education level, living arrangement (living alone or with a partner), comorbidity, eye condition (e.g. macular degeneration, diabetic retinopathy, glaucoma), ear condition (e.g. presbycusis, tinnitus) and hearing acuity (speech-reception-threshold in noise, defined as the signal-to-noise ratio corresponding to 50% intelligibility) will be collected by research assistants. Visual acuity and other relevant data, such as the eye condition, will be obtained from the patient’s files at the low vision rehabilitation centers with the patient’s consent. *Cognitive impairment* is assessed with the six-item screener derived from and comparable to the full Mini-Mental State Examination (MMSE) [78]. Finally, the Dutch version of the Center for Epidemiological Studies-Depression scale (CES-D), a general indicator of *depressive mood*, is used to assess the presence of depressive symptoms, which may influence the outcome of the study [79]. Participants will be asked if major life events have occurred between baseline and follow-up.

#### Communication partner

The HHDI ‘reactions of others’ scale assesses attitudes towards the hearing impaired partner and has been used to measure *coping with hearing loss*[80]. *Self-reported change from hearing aids* and *self reported change from communication strategies* reported by the communication partner will be measured with the Dutch versions of the International Outcome Inventory for hearing aids (IOI-HA-SO) and the International Outcome Inventory for alternative strategies (IOI-AI-SO) [67]. *Health* was measured as subjective health and with the EuroQol 5 Dimensions (EQ-5D) [69]. *Evaluation of the DSL protocol* with questions: “Are you satisfied with the advice of the OT?”, “Are you satisfied with the treatment?”, and ”Are you satisfied with the results?”. *Chronic fatigue* was assessed with the Fatigue Assessment Scale (FAS) [74]. *Self-efficacy* is measured with the General Self-Efficacy Scale (GSES) divided in three subscales: Initiative, Effort and Persistence [82]. *Depressive symptoms* will be assessed with the Dutch version of the Center for Epidemiological Studies-Depression scale (CES-D) [79].

#### Costs

In a bottom-up price calculation all costs (intervention, health care costs, costs of formal and informal care) will be calculated for both the intervention and control group. Intervention costs include participation of the OT (time, travel time and expenses), participation of the communication partner (time, travel time and expenses). Healthcare costs include costs of medication and consultations of health care providers. The volume of will be measured with the iMTA Medical Consumption Questionnaire (iMCQ) [83] and costs will be evaluated according to the prices suggested in the guidelines for economic evaluation in The Netherlands [86]. If guidelines are not available, costs will be estimated.

#### Statistical analysis

Data will be analyzed according to the intention-to-treat principle. Linear mixed models for continuous outcomes and generalized linear mixed models for counts and categorical outcomes will be used to assess treatment effects with respect to primary and secondary outcome measures. Treatment effects will be assessed according to the analysis strategy as described by Winkens et al. [87-89] and is defined as the treatment allocation*time interaction. To account for correlatedness of outcomes within the same therapist, a random intercept for therapist is included in the model.

For participants who are not treated according to the protocol, intention-to-treat analyses will be compared to per-protocol analyses. Data will be analyzed using the software package SPSS 20 for Windows.

#### Sample size

Power calculations are based on expected progress in use of communication strategies (the Communication Strategies Scale of the Hearing Handicap and Disability Inventory), which has been the primary outcome of previous studies in persons with hearing loss [41]. In a previous RCT by Kramer et al. (2005) on the effectiveness of a home education program for older adults with hearing impairment only, the mean difference in communication skills between the intervention and control group was about 0.5 (SD = 0.8) [41]. Sample size calculations of this RCT are based on a linear mixed models and confirmed by some simulations (data not shown). Sixty-two participants per arm, with adjustment for clustering by 8 therapists in the intervention condition, provide a power of 0.80 (1-β) with alpha 0.05 (two-sided significance level), to detect a 0.5 difference between trial arms after 3 months (corrected for the differences at baseline), after taking into account a 20% dropout rate.

#### Economic evaluation

In addition to the RCT, an economic evaluation will compare costs and consequences from a societal perspective of an intervention group receiving the DSL protocol compared with a waiting list control group. Therefore, all costs and consequences of the DSL protocol will be taken into account for patient, communication partner and society. The incremental cost-effectiveness ratios (ICER) will be calculated; the difference in mean costs between intervention and control group will be divided by the difference in outcome measures between the two groups. Because costs data are generally skewed, non-parametric bootstrapping with 5000 replications of both intervention and control group will be used to derive 95% confidence intervals for the ICER. Bootstrapped cost-effectiveness pairs will be plotted in a cost-effectiveness plane and cost-effectiveness acceptability curves will be estimated [90].

## Discussion

Especially among visually impaired elderly, dual sensory loss (DSL) is highly common. Of all the related difficulties, communication is perhaps the most challenging and it may negatively affect a patient’s health and wellbeing. We expect the newly developed DSL protocol to reduce these difficulties. This may lead to better hearing aid use, improved use of effective communication strategies and hence, better quality of life, health and wellbeing. This paper describes the ‘Dual Sensory Loss-protocol’ and the design of a multicenter international RCT to determine the effectiveness and cost-effectiveness of the DSL protocol.

In the development of the DSL protocol, designed for OTs working in low vision rehabilitation, we obtained information from the literature, which we complemented with interviews and discussions with patients and professionals (working in ear and eye care). The trial will test the effectiveness of the additional DSL protocol compared to a waiting list control group on use and maintenance of hearing aids; communication; coping with a dual sensory impairment; social participation and quality of life of the patient and communication partner; and cost-effectiveness from a societal perspective.

The development of the protocol and design of the RCT required decisions as to which professionals would be most suitable to perform the protocol, and which DSL patients should be included in the trial.

Firstly, the DSL protocol consists of three chapters suitable for different rehabilitation professionals. On the one hand, the first two chapters of the DSL protocol focus on maximizing use of the senses with the use of hearing aids; other assistive devices; and minor adaptations to the living environment; these are considered highly suitable topics to be handled by OTs. On the other hand, the last chapter focuses on psychosocial issues: it discusses communication difficulties, psychosocial problems, coping with dual sensory impairment, and also teaches communication strategies; some consider that these topics are more suitable for social workers. To be able to build a relationship of trust, the patient can best be handled by one professional, and we decided OTs are the most competent. Secondly, we decided to recruit DSL patients who already received usual low vision and audiology care, i.e. patients who possess hearing aids and who have received low vision rehabilitation. This allows us to investigate the added value of the DSL protocol compared to a waiting list control group (which was allowed to receive other interventions if needed).

Several studies have aimed to meet the urgent need for evidence-based protocols and interventions in rehabilitation [91-94]. However, until now, little attention has been paid to the development and evaluation of interventions for the vulnerable group of DSL patients, who represent an urgent research need [47]. Our innovative study on rehabilitation of DSL for use in low vision rehabilitation is one of the few addressing these needs in older patients with age-related DSL. Additionally, low vision patients who seek help for their impairment at multidisciplinary low vision rehabilitation centers will likely be open to rehabilitation in general. We believe our DSL protocol will assist frail elderly with DSL in low vision rehabilitation; it addresses urgent needs not yet addressed by other interventions.

However, there are limitations to the study concerning both the protocol and the RCT. First, the DSL protocol was developed for patients with some residual vision and hearing, which concerns the vast majority of DSL patients [95], and focuses on maximum use of both senses. Therefore, the protocol is less suitable for totally blind and/or deaf patients; information on teaching tactile sign language is not incorporated. Also, although we believe that the DSL protocol is comprehensive and includes various forms of rehabilitation, eccentric viewing is not included. It maybe worthwhile for future implementation of the protocol to include eccentric viewing strategies to improve speech reading in patients with central scotoma [38]. Other limitations are related to the choice of a pragmatic instead of an explanatory trial. Further standardization of the DSL protocol would increase the ability to adequately evaluate the effectiveness. Standardization of the protocol could be improved by, e.g. standardizing the exact amount of time per exercise and chapter, and the number of sessions per patient. However, in daily practice it is very important to adapt to the needs of the individual patient, e.g. severity of vision and hearing impairment; or other impairments/limitations due to comorbidity, learning abilities, fatigue or concentration. For this reason, the current DSL protocol is adaptive to suit the needs of the individual patient. In line with the suboptimal standardization, the rather heterogeneous study population could be another limitation. However, to ensure generalizability, the study population had to reflect the variations among patients which occur in actual rehabilitation practice and to best represent patients in whom the treatment would be applicable.

Second, due to budgetary restrictions it was not possible to provide information on the long-term effects. Third, blinding of participants and OTs is not possible since no placebo treatment is included in the study to account for the placebo effect. Participants may report change as a result of simply meeting with an empathetic professional each week to discuss problems. Therefore, the effect of the DSL protocol is the total difference between groups, including both treatment and associated placebo effects. This has both advantages and disadvantages: a disadvantage is that the pure effect of the DSL protocol’s content remains unclear whereas, on the other hand, reality is best reflected. This pragmatic trial provides the best reflection of the likely rehabilitation outcome in actual practice.

This study provides useful information on DSL. Also, if the trial shows the DSL protocol to be effective, this will allow multidisciplinary low vision rehabilitation centers to provide an evidence-based treatment protocol for DSL patients. The DSL protocol will be an important tool for OTs to assist their older patients with DSL in the use of hearing aids, to maximize use of the senses, and to teach patients and/or communication partners specific skills to improve communication.

However, DSL needs more attention in other care settings (besides low vision rehabilitation), such as nursing homes and audiology rehabilitation. It is estimated that about 2% of the elderly who consult a hearing healthcare professional experience such visual impairment to such extent, that it limits the perception of facial cues for communication [54]. Although future research on DSL in audiology care is recommended, rehabilitation of DSL in the setting of audiology care requires even more effort. Hearing impairment in the elderly occurs much more frequently than visual impairment. Therefore, DSL in audiology rehabilitation is less common, so that more patients need to be screened to detect patients with DSL. In addition, low vision and hearing rehabilitation is organized in different ways. For example, in the Netherlands, many older adults with hearing loss in the Netherlands do not consult a multidisciplinary audiology rehabilitation center but go directly to a hearing aid dispenser; this occurs much less with low vision.

Besides special treatments for DSL, there is a need for more collaboration between low vision and audiology rehabilitation by, for example, making greater use of referrals [53]. To facilitate this, rehabilitation professionals working in low vision and audiology need interdisciplinary training, to enable them to detect problems associated with DSL and to refer patients as required.

In conclusion, until now, insufficient attention has been paid to the problems of elderly with DSL. However, the development of this DSL protocol represents an important step to improve the health and quality of life of DSL patients.

## Abbreviations

DSL: Dual sensory loss; HA: Hearing aid; OT: Occupational therapist; RCT: Randomized controlled trial.

## Competing interests

The authors declare that they have no competing interests.

## Authors’ contributions

RvN conceived and designed the study and GvR, JF SK and DL advised on the study and its design. HV developed a draft of the DSL protocol and GvR, SK, JF RvN helped to draft and revise it. DL performed the power calculation of the RCT and HV conducts the trial. HV drafted the manuscript, which was revised by all other authors. All authors have read and approved the final version to be published.

## Pre-publication history

The pre-publication history for this paper can be accessed here:

http://www.biomedcentral.com/1471-2318/13/84/prepub
